# Deep learning-based quantification of NAFLD/NASH progression in human liver biopsies

**DOI:** 10.1038/s41598-022-23905-3

**Published:** 2022-11-10

**Authors:** Fabian Heinemann, Peter Gross, Svetlana Zeveleva, Hu Sheng Qian, Jon Hill, Anne Höfer, Danny Jonigk, Anna Mae Diehl, Manal Abdelmalek, Martin C. Lenter, Steven S. Pullen, Paolo Guarnieri, Birgit Stierstorfer

**Affiliations:** 1 Drug Discovery Sciences, Boehringer Ingelheim Pharma GmbH & Co. KG, 88397 Biberach an der Riß, Germany; 2 Cardiometabolic Diseases Research, Boehringer Ingelheim Pharmaceuticals, Inc., 900 Ridgebury Road, Ridgefield, CT 06877 USA; 3 Global Computational Biology and Digital Sciences, Boehringer Ingelheim Pharmaceuticals, Inc., 900 Ridgebury Road, Ridgefield, CT 06877 USA; 4 Institute of Pathology, Hannover Medical School, and the German Center for Lung Research (DZL), Biomedical Research in Endstage and Obstructive Lung Disease Hannover (BREATH), 30625 Hanover, Germany; 5 Duke Department of Medicine, Gastroenterology, Lasalle Street, GSRB 1, Durham, NC 27710 USA; 6 Non-Clinical Drug Safety, Boehringer Ingelheim Pharma GmbH & Co. KG, 88397 Biberach an der Riß, Germany

**Keywords:** Diagnostics, Computer science, Metabolic syndrome, Outcomes research, Liver cirrhosis, Liver fibrosis, Non-alcoholic fatty liver disease, Non-alcoholic steatohepatitis

## Abstract

Non-alcoholic fatty liver disease (NAFLD) affects about 24% of the world's population. Progression of early stages of NAFLD can lead to the more advanced form non-alcoholic steatohepatitis (NASH), and ultimately to cirrhosis or liver cancer. The current gold standard for diagnosis and assessment of NAFLD/NASH is liver biopsy followed by microscopic analysis by a pathologist. The Kleiner score is frequently used for a semi-quantitative assessment of disease progression. In this scoring system the features of active injury (steatosis, inflammation, and ballooning) and a separated fibrosis score are quantified. The procedure is time consuming for pathologists, scores have limited resolution and are subject to variation. We developed an automated deep learning method that provides full reproducibility and higher resolution. The system was established with 296 human liver biopsies and tested on 171 human liver biopsies with pathologist ground truth scores. The method is inspired by the way pathologist's analyze liver biopsies. First, the biopsies are analyzed microscopically for the relevant histopathological features. Subsequently, histopathological features are aggregated to a per-biopsy score. Scores are in the identical numeric range as the pathologist’s ballooning, inflammation, steatosis, and fibrosis scores, but on a continuous scale. Resulting scores followed a pathologist's ground truth (quadratic weighted Cohen’s κ on the test set: for steatosis 0.66, for inflammation 0.24, for ballooning 0.43, for fibrosis 0.62, and for the NAFLD activity score (NAS) 0.52. Mean absolute errors on a test set: for steatosis 0.29, for inflammation 0.53, for ballooning 0.61, for fibrosis 0.78, and for the NAS 0.77).

## Introduction

Non-alcoholic fatty liver disease (NAFLD) refers to a spectrum of liver conditions ranging from simple fat accumulation (steatosis) to non-alcoholic steatohepatitis (NASH). NAFLD has a global prevalence of approximatively 24% in adults^1^. Frequent risk factors for NAFLD are obesity^1^ and diabetes^2^; or more generally, features of the metabolic syndrome^1^. Accumulation of triglyceride in hepatocytes, termed hepatic steatosis, is considered an early event in the pathogenesis of NAFLD. Hepatic steatosis can develop into concurrent inflammatory liver injury, referred to as NASH. It is estimated that 20% of patients with NAFLD had NASH in 2015^3^. Older age has been identified as a risk factor for NASH and thus, estimates for 2030 predict that the fraction of NAFLD patients with NASH will increase to 27% due to population aging^3^. Because NASH can lead to cirrhosis^4,5^ and the development of hepatocellular carcinoma (HCC) (6), it is a major cause of liver-related morbidity and mortality. Indeed, NAFLD is now driving the increased social and economic burden of liver disease in many countries, as evidenced by the fact that it is the fastest growing indication for liver transplantations in the United States^6^. Currently the only treatment option for NAFLD/NASH are lifestyle changes. Despite the unmet medical need, no supporting approved drug therapy is available^7^.

The gold standard for NAFLD/NASH diagnosis and assessment of the disease severity currently involves a tissue sample (e.g., a liver biopsy) and microscopic inspection of stained slides by a pathologist^8^. Various histopathological scoring systems are used to quantify disease severity of NAFLD/NASH^9^. The system developed by Kleiner, Brunt and the NASH Clinical Research Network (CRN) pathology committee is frequently used^10^. In this system, ballooning, inflammation, and steatosis—three histological features of active injury of the liver—are analyzed and combined as NAFLD activity score (NAS)^10^. In addition, the less reversible but diagnostically and pathologically important feature fibrosis is analyzed as a fourth feature. For each of the features, a discrete score is assigned by a pathologist according to the presence, morphology, density, and localization of their respective histological hallmarks.

This manual scoring procedure has several drawbacks. First, the resolution is limited. Kleiner’s steatosis score has only four distinct values, ballooning three, inflammation four, and fibrosis five, although the underlying biological states are typically continuous. Second, histopathological liver scoring requires a trained pathologist, an in-demand occupation^11^. Third, there is substantial intra- and inter-operator variability between the scores when asking different pathologists or the same pathologist at different times^10^. Hence, comparability and precision of the results is limited. Automated systems would enable more comparable, precise, and rapid assessment of NAFLD/NASH.

Deep learning-based approaches have recently achieved important breakthroughs in image analysis. In 2012, a convolutional neural network (CNN, a form of deep learning) won the ImageNet image classification challenge by a such a substantial margin that deep learning-based approaches introduced a paradigm shift in automated image analysis^12^. Today, in many tasks CNNs are superior to classical computer vision algorithms (e.g., image classification^12^). Given enough training data, CNNs can also outperform humans^13^. In digital histopathology, applications are numerous^14^ and encompass quantification of disease progression in animal models^15^, prediction of disease progression after liver cancer surgery^16^, prediction of immune gene signatures from liver histology^17^, and staging, grading and classification of human diseases, such as various cancers^18,19^, or liver disease^20^. We expect that an increasing number of image analysis tasks in histopathology are about to be automated.

In this study, we present an open source, ‘Artificial Intelligence’ AI solution for obtaining the Kleiner and Brunt scores that addresses the shortcomings of the current pathologist-dependent scoring approaches, mentioned above. The method automatically quantifies the components of the Kleiner and Brunt scores by analyzing microscopy images of human liver biopsies. In brief, the method analyzes liver samples like a pathologist: First it locally analyzes the tissue for relevant features. It then combines the distributed local features for the whole tissue to obtain per-tissue scores. We use Masson Trichrome and Masson Goldner collagen stains to train our system, however the method is generalizable to other stains like Hematoxylin and Eosin (H&E), or H&E combined with Trichrome stains. Our aim to create an automated algorithm to quantify the morphological characteristics of independently diagnosed NAFLD/NASH (e.g., after differential diagnosis) and to measure the severity of alterations in a histological liver sample. Resulting scores are in the same numeric range as the features of the Kleiner and Brunt score (e.g., 0–4 for fibrosis), but with continuous scaled outputs. This increased precision has the potential to differentiate between more subtly nuanced disease states, which can be helpful in combination with other data sets or to measure treatment success. The calculated results are reproducible and do not exhibit variability, e.g., when the same biopsy is analyzed a second time, as is the case with pathologists. This work improves our previously published system analyzing features of NASH in animal models^15^. We share the relevant data and source code, with the aim to stimulate rapid progress in applying deep learning-based approaches to medicine and drug discovery. The approach can be extended with custom training data to iteratively match a wider variety of stains, scanners, patients, and pathologist scores for an improved assessment of NAFLD/NASH progression.

## Results

**Figure 1:**
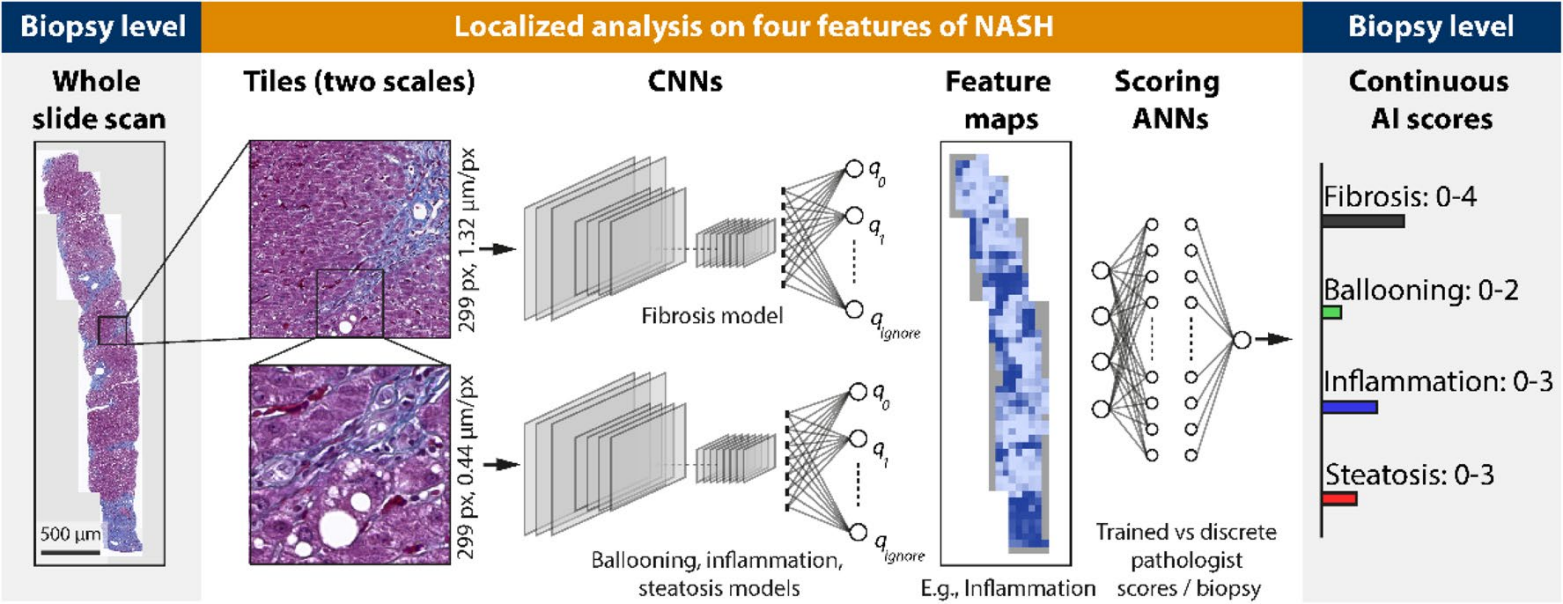
Workflow to analyze human liver biopsies to obtain pathologist-like NALFD/NASH scores*.* A whole slide scan of a liver biopsy is analyzed by four dedicated convolutional neural networks (CNNs), which classify the features of interest (fibrosis, ballooning, inflammation, steatosis). The classification is done using a grid of image tiles generated at two scales, reflecting the different dimensions of the features of interest: for fibrosis ‘low resolution’ tiles are used, whilst for ballooning, inflammation, and steatosis ‘high resolution’ ones. The CNN’s classification result in histopathological feature maps showing how the features distribute over the tissue. Distributed features are then aggregated to a form a single score, comparable to what a pathologist would do it when assigning scores after analyzing a biopsy microscopically. This aggregation is done using an artificial neural network (ANN) trained to match the pathologist scores provided as ground truth. As result continuous scores on the four features of the Kleiner and Brunt score are obtained.

Figure 1 gives an overview of our workflow for determining the pathologist-like scores from biopsies. We use digitized whole slide scans of human liver biopsies as input (left). The biopsies are analyzed tile-by tile by dedicated CNNs in a grid covering the whole biopsy. Ballooning, inflammation, and steatosis are analyzed at a magnification of several cells (‘high resolution tile’, side length 132 µm), whereas fibrosis is analyzed at the mesoscopic tissue level (‘low resolution tile’, side length 395 µm). As a result, histological feature maps are created, which encode the spatial distribution of relevant features over the biopsy (e.g., spatial pattern of inflammation). Next, we aggregate the spatially distributed feature maps to obtain a single score value per biopsy on each of the four features ballooning, inflammation, steatosis, and fibrosis (right). Traditionally this step is done by a pathologist, who analyzes the sample microscopically at different areas and aggregates to a score per biopsy, based on training and experience. In this study, the aggregation is done by dedicated scoring artificial neural networks (ANNs). The ANNs used the histological feature maps as input and produce scores that were optimized to closely match a ground truth of pathologist scores. We engineered the ANNs such that the resulting scores are bound to the same numerical range as the pathologist scores and therefore possess the identical interpretation in their numerical values. It is important to note that our system is not restricted to a discrete classification in the disease state but is free to choose values on a continuous scale. This approach can thus provide a higher resolution, which may help in a precise assessment of disease progression.

In the following, details of our newly developed method are described and first experimental results from its application are reported.

**Figure 2:**
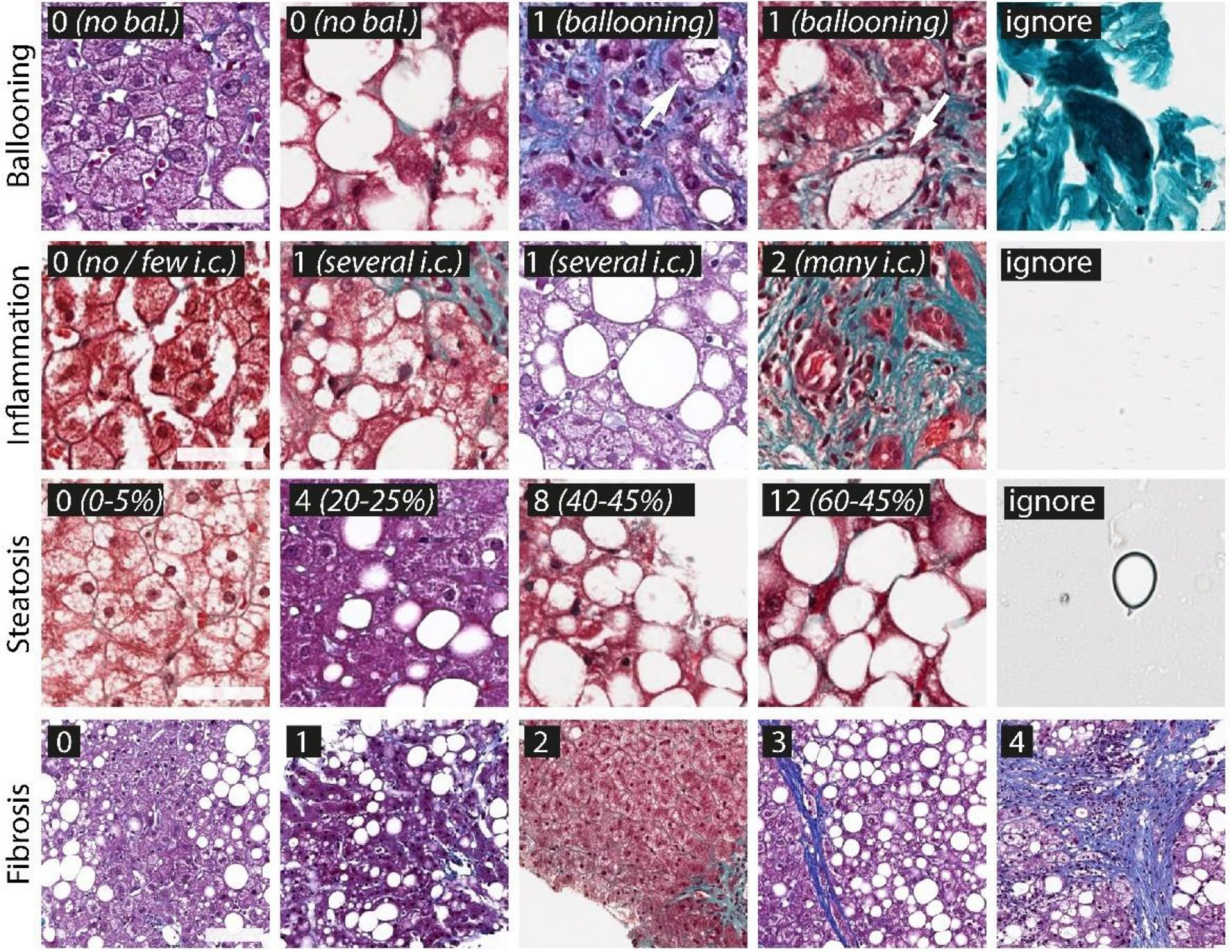
Overview of the training classes of the four CNN models. Models are shown in rows and examples of the classes shown in columns. Class descriptions are shown on the top left of the tiles. For ballooning classes ‘0’ and ‘1’ were defined, with ‘0’ defined by the absence of a ballooning cell and ‘1’ by the presence of a ballooning hepatocyte (arrows). In case of inflammation, classes ‘0’, ‘1’ and ‘2’ were defined depending on the density and morphology of inflammatory cells visible on a tile. For steatosis, class bins of a width of 5% were defined (not all classes shown). For fibrosis the classes per tile corresponded to the macroscopic definition of the fibrosis scores. Each model contained an ignore class to identify artifacts or any kind of non-liver structure (not shown for fibrosis). Scale bars first of three rows (‘high resolution tiles’): 50 µm, scale bar of last row (‘low resolution tiles’): 100 µm.

The first step in our automated Kleiner and Brunt scoring system is the accurate recognition of histopathological features on the microscopic level. This requires trained image-classification CNNs. For this purpose, we labelled image tiles for training and validation (see “Materials and methods” for details). The total set of image tiles comprised a large pool of slides that originated from three sources, two different whole slide scanners and were stained with two variants of Masson’s stain (Masson Trichrome and Masson Goldner). We intentionally opted for this broad variability in the dataset, as variability of training data frequently results in more robust algorithms^21^. Figure 2 gives an example on the four models (rows) and the defined classes (columns) within each model (i.e., histological feature to be classified).

We developed a custom tool to speed up labelling and use the time of the annotator more efficiently; the LabelTool shown in Supplementary Fig S2. This tool allowed to optimize the annotate—train—review cycle by analyzing individual tiles while at the same time displaying the adjacent tiles and thus providing the context of each slide to the human expert. Moreover, the tool allowed to visualize classification results from a current classification iteration. This approach helped to focus the training data generation on the most challenging cases, i.e., perform active learning^22^.

**Figure 3:**
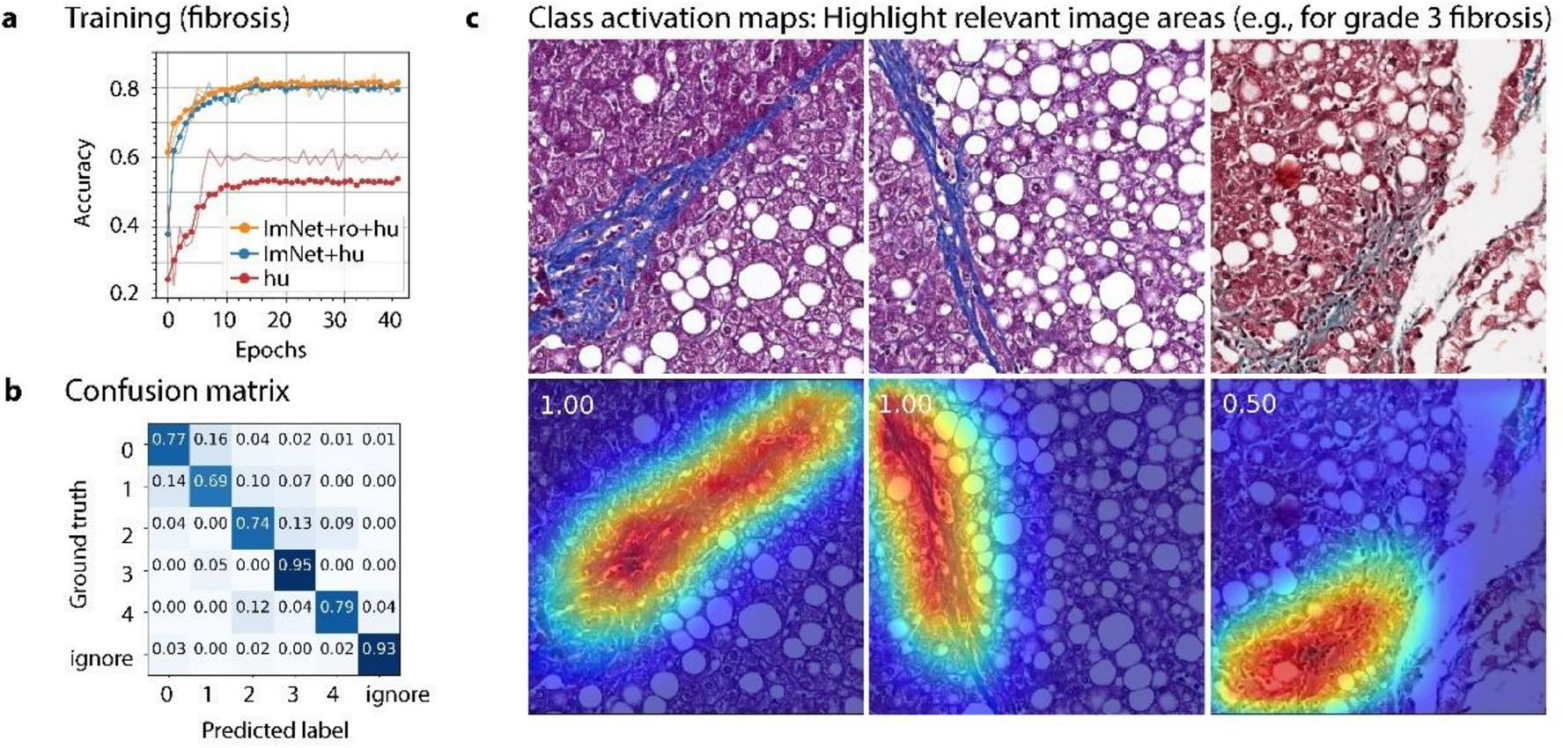
Training, quantitative evaluation, and qualitative visualization of CNN’s decisions in example of the fibrosis model. (**a**) Learning curves of the fibrosis CNN trained under three different pre-training conditions. All learning curves show an increase of accuracy with the training iterations (epochs) followed by a plateau where the final accuracy is reached. Using no pretraining and only the annotated human fibrosis tiles (‘hu’) resulted in relatively poor final accuracy of 61.0%. Prior pre-training with ImageNet (‘ImNet + hu’) resulted in a final accuracy of 79.4%. Additional pretraining with available domain-related data from rodent liver fibrosis (‘ImNet + ro + hu’) resulted in slightly faster learning and marginally better final model performance. Bold lines are performances on the validation set, thin lines on the training set. (**b**) A confusion matrix compares the CNN classifications on unseen validation tiles with the ground truth from the pathologist. (**c**) Class activation maps (CAM) allow showing relevant regions for the decision of a CNN for a certain class. Here, the activations for the fibrosis model and grade 3 fibrosis (bridging fibrosis) are shown. The number at the top left of the CAM indicates the CNNs confidence for grade 3 fibrosis. The right column shows a case with more ambiguity.

Figure 3 shows results of training and evaluation of the CNN models in the example of fibrosis. To find the most effective training approach, we compared three different transfer learning approaches^23^ (Fig. 3a). CNN pretraining on ImageNet turned out to be crucial four our moderately sized dataset, as shown by a validation accuracy of 61.0% without pretraining compared to 79.4% when pretrained on ImageNet. A second pretraining experiment used a CNN model that was pretrained on ImageNet and on the identical features of rodent NASH^15^. This resulted only in a marginally better final validation accuracy of 80.4%. In practice this small improvement might not justify the additional effort. For all four CNNs, the confusion matrix confirmed a very good classification performance of recognizing the relevant histological features over all classes (Fig. 3b for fibrosis as an example). See supplementary material for further details on transfer learning and evaluation results.

Another approach to investigate how CNNs make decisions are class activation maps^24^, shown in Fig. 3c. This method allows highlighting the relevant regions for the decision of a CNN, e.g., for score 3 fibrosis. An advantage is that class activation maps allow to ensure that indeed the structures of interest lead to a classification decision and no other possibly confounding histological features. The example in Fig. 3C shows class activation maps for the fibrosis model and grade 3 fibrosis and confirms that elongated fibrotic streaks are used for the CNNs decision, the correct morphological structures for bridging fibrosis.

**Figure 4:**
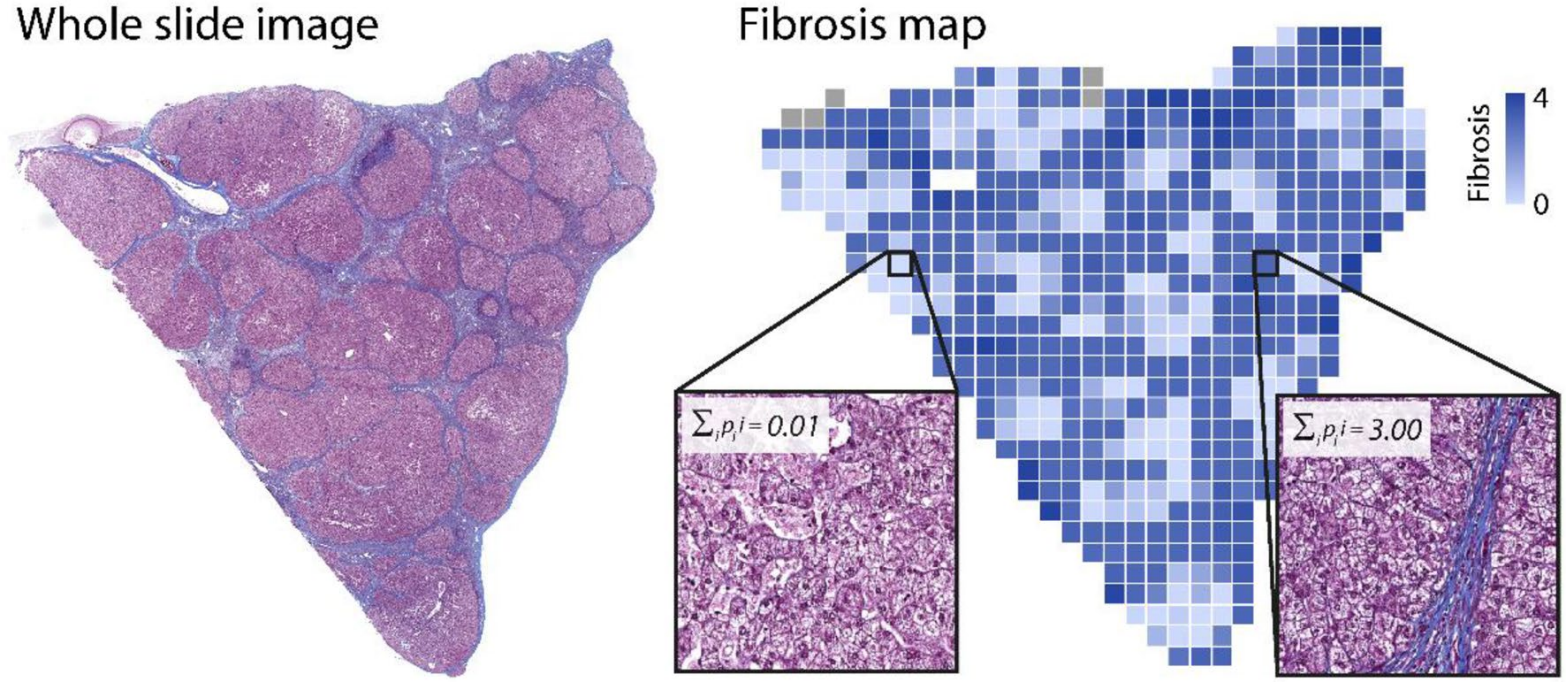
Whole slide image of a liver sample and corresponding feature map of fibrosis. Left: Whole slide image of liver tissue stained in Masson Trichrome. Applying the trained CNN to the whole slide image yielded tile-by-tile results in in the form of a fibrosis feature map, shown at the right. The heatmap was generated using the software Spotfire Analyst 10.10.3 (https://www.tibco.com/products/tibco-spotfire, Tibco Software Inc., Palo Alto, USA). The two insets at the right illustrate example classifications and the weighted fibrosis score of these two tiles.

Application of the trained CNN models to tissue allowed to obtain maps showing the spatial distribution of histological features. Figure 4 shows an example of the fibrosis feature map for a sample of human liver tissue.

**Figure 5:**
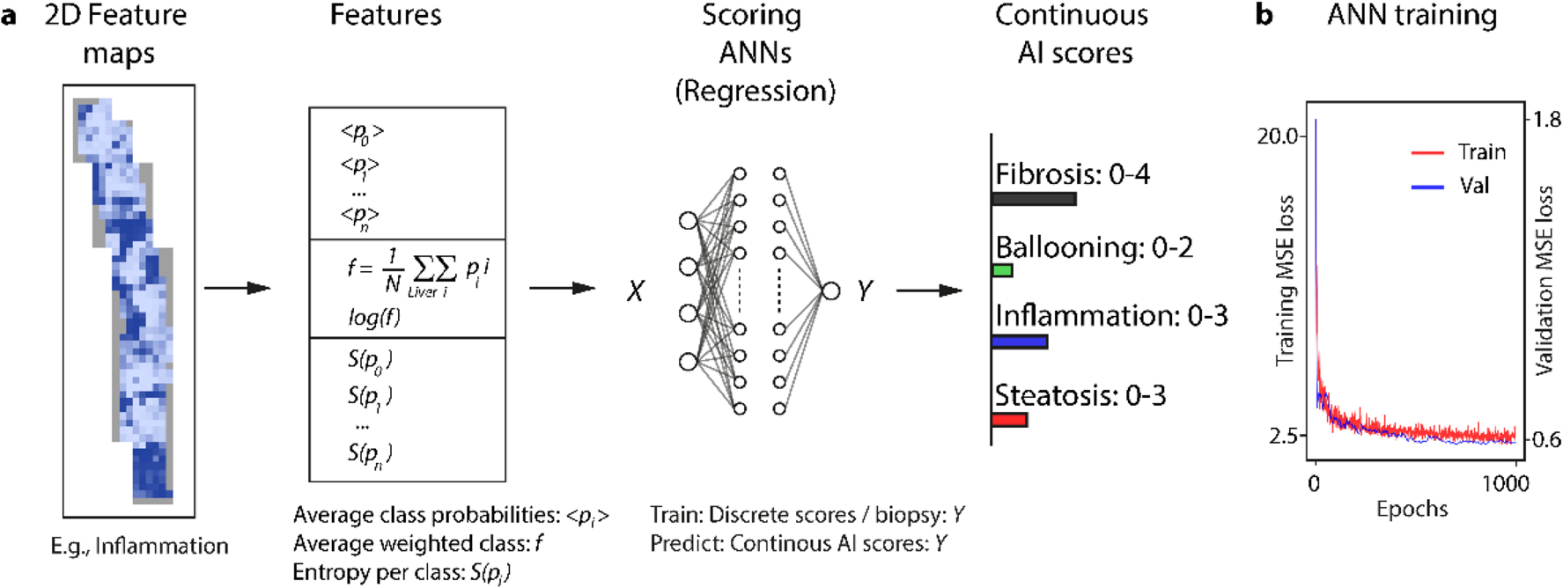
Aggregation of 2D histological feature maps to obtain continuous AI-based Kleiner & Brunt scores. (**a**) For each score distributed features (left) are converted into average class probabilities, average weighted classes, and entropies per class (second column from left). These values are used as input for a dedicated ANN that trained to predict the pathologist scores from the input with minimized deviation (third column from left). The ANN output has the same range as the Kleiner and Brunt score and is therefore directly interpretable. In contrast to scores from human experts, it generates continuous-scaled outputs, for example to account for transitions two discrete scores. (**b**) Training curves of the ANN show the decrease of the mean squared error (MSE) loss during training. The MSE quantifies the deviation of pathologist ground truth and ANN predictions.

Next, we converted the distributed histological features into a single pathologist-like score per biopsy, as illustrated in Fig. 5. From the distributed histological feature maps that were generated by the CNNs, aggregated features such as the average and entropy of the classes were computed, characterizing the distribution of the respective classes. Scoring-artificial neural networks (ANN) were engineered (i.e., one ANN for ballooning, inflammation, steatosis, and fibrosis), using pathologist’s scores as ground truth. These scoring-ANNs use the features like average and entropy as input to predict the respective score. Thus, the ANNs learned what traditionally pathologists do, when microscopically analyzing a biopsy and mentally aggregating to a score per sample. The learning curve confirmed a good fit to the data, with minimized mean squared error (MSE) between ANN-scores and pathologist scores (ground truth). The output of our ANN was engineered to the range of the pathologist scores, however without the restriction of discrete scores. The resulting continuous score is thus able to exhibit intermediate values and consequently has a higher resolution of the disease state.

**Figure 6:**
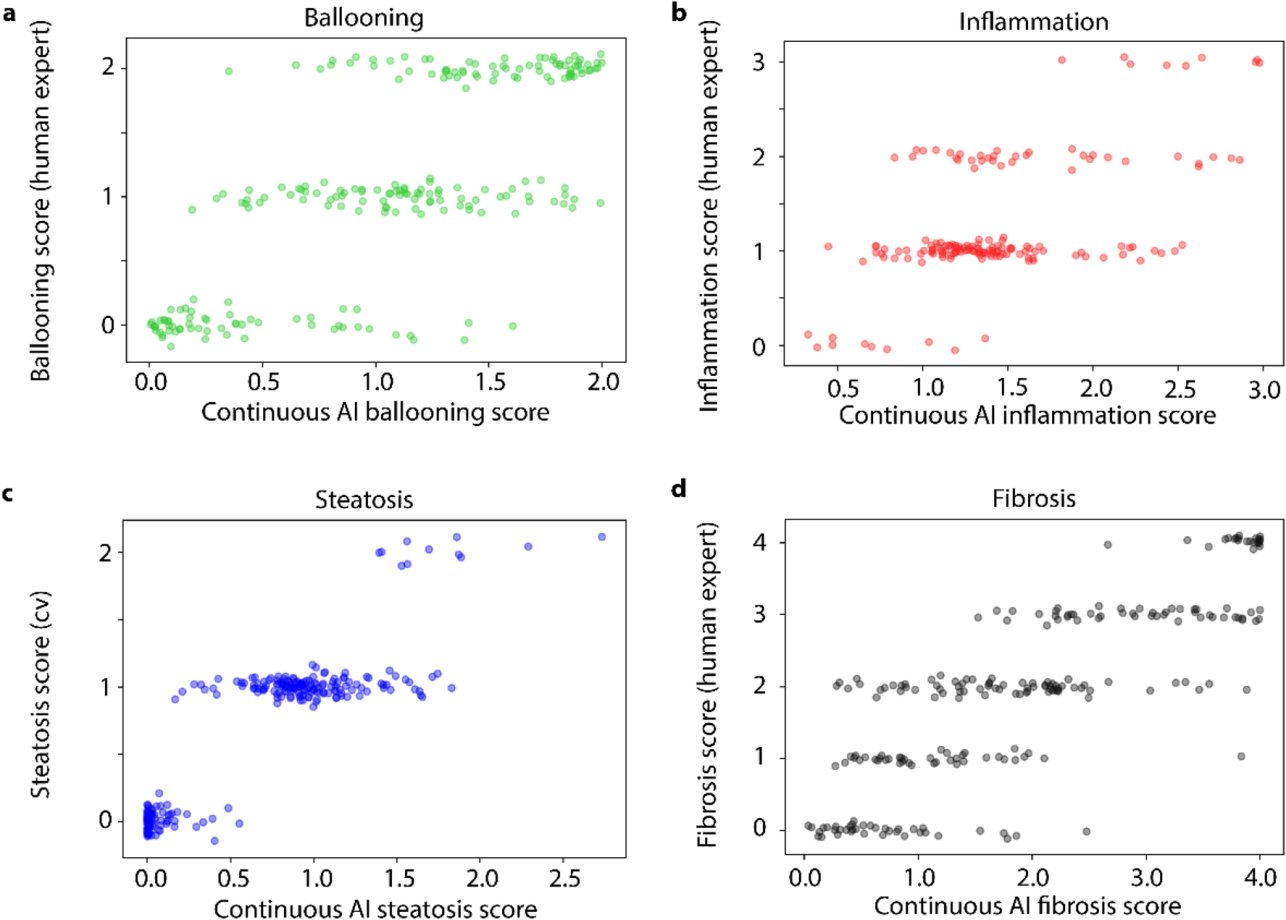
Continuous AI scores vs. discrete ground truth scores. Results for (**a**) Ballooning, (**b**) inflammation, (**c**) steatosis, and (**d**) fibrosis. AI scores on the horizontal axis are results from ANN fits on unseen validation data (four-fold cross validation). The AI scores cover the same range as the pathologist scores but are continuous. Jitter was added to the discrete pathologist scores for visualization purposes. The steatosis ground truth is a corrected score from a separate computer vision analysis of steatosis the slides, since pathologists systematically overestimate steatosis scores as shown in Fig S1. Our corrected scores were exclusively in the range 0–2.

Figure 6 shows results of the scoring-ANNs compared to the ground truth scores. The scoring ANNs provide values in the identical range as the pathologist scores, but these values distribute along the whole range of possible values. The step like pattern shows the overall agreement of ground truth scores and ANN-scores. To measure the expected average deviation of ground truth and AI score, we quantified the performance of the ANN fit with the mean absolute error (MAE) by using 4 × cross validation on set 1 and on the test slides (set 2). For ballooning, we obtained an MAE = 0.40, (MAE = 0.58 on the test set). For inflammation MAE = 0.47 (MAE = 0.53 on the test set). Steatosis resulted in MAE = 0.16 (MAE = 0.29 on the test set) and fibrosis in MAE = 0.57 (MAE = 0.76 on the test set). For the NAS an MAE = 0.60 (MAE = 0.75 on the test set) was obtained. In addition, we quantified the quadratic weighted Cohen’s κ, which is a variant of Cohen’s κ which considers the magnitude of deviation of the prediction and the pathologists ground truth. Here, results with 4 × cross validation on set 1 were: steatosis κ = 0.89 (0.66 on the test set), inflammation κ = 0.54 (0.24 on the test set), ballooning κ = 0.60 (0.43 on the test set), fibrosis κ = 0.82 (0.62 on the test set), and NAS κ = 0.55 (0.52 on the test set). The increase in the MAE and quadratic weighted Cohen’s κ on the test set compared to the result from cross validation showed a slight over adaption to set 1. An overview of quantitative evaluation results per slide are shown in Table 1.

**Table 1:** Deviation of pathologist scores and AI scores. Performance metrics of scores per slide on set 1 (train set, using fourfold cross validation (4 × cv)) and on set 2 (test set). Note that only the two first performance metrics mean absolute error and quadratic weighted Cohen’s κ take into account the magnitude of the deviation. Weighted average scores are the support-weighted averages per numeric class.

Score	Dataset	Mean absolute error	Quadratic weighted Cohen's κ	Weighted average precision	Weighted average recall	Weighted average F1	N
Steatosis	Set 1 (train, 4 × cv)	0.16	0.89	0.94	0.92	0.93	277
Steatosis	Test	0.29	0.66	0.89	0.82	0.85	170
Inflammation	Set 1 (train, 4 × cv)	0.47	0.54	0.64	0.62	0.63	160
Inflammation	Test	0.53	0.24	0.61	0.51	0.54	75
Ballooning	Set 1 (train, 4 × cv)	0.16	0.60	0.64	0.63	0.63	206
Ballooning	Test	0.29	0.43	0.68	0.47	0.46	146
Fibrosis	Set 1 (train, 4 × cv)	0.57	0.82	0.62	0.56	0.55	218
Fibrosis	Test	0.76	0.62	0.42	0.38	0.37	146
NAS	Set 1 (train, 4 × cv)	0.60	0.55	0.55	0.54	0.54	146
NAS	Test	0.75	0.52	0.44	0.43	0.42	67

**Figure 7:**
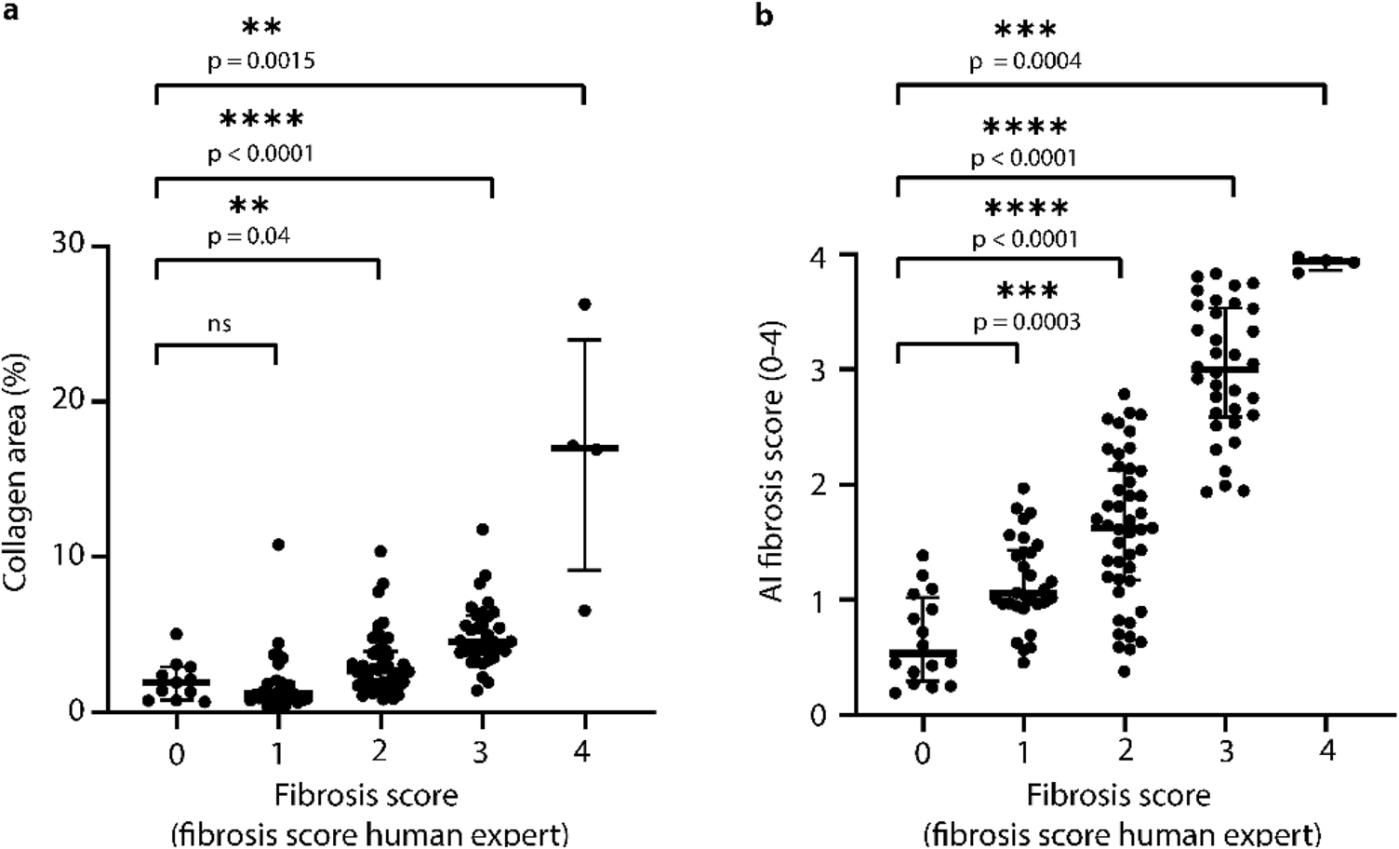
Comparison of fibrosis quantification using ‘classical computer vision’ and the AI fibrosis score. (**a**) Analysis with ‘classical computer vision’. The collagen area (%) obtained from analyzing the collagen specific color component of Masson’s Goldner stain shown on the vertical axis compared to the pathologist’s fibrosis scores. For the more severe fibrosis scores 2, 3 and particularly 4 collagen area is significantly elevated compared to the control (fibrosis score 0). The initial changes (fibrosis scores 1 and 2) result in a no or a moderate increase in collagen area. (**b**) With the AI fibrosis score, all levels of fibrosis can be discriminated significantly from the control (score 0). The difference in resolution can be explained by the different working mechanisms. The AI-based detection can be trained to recognize differences in morphological structures before they markedly impact area (%), e.g., sinusoidal collagen. AI scores are from validation data of 4 × cross validation.

Finally, in the case of fibrosis, we compared how results from ‘classical image analysis’ (collagen area based on color) and the deep learning-based fibrosis score relate to the pathologists scores (Fig. 7). With ‘classical image analysis’, score 0 and 1 fibrosis could not be distinguished since the features of score 1 fibrosis do not markedly affect collagen area. For the higher fibrosis scores, a discrimination was possible since these stronger fibrotic degrees start markedly affecting area. Especially stage 4 fibrosis (cirrhosis) resulted in substantially elevated collagen areas. In comparison, the AI-fibrosis score could separate also initial fibrotic changes such as a score of 1 from a score of 0. The separation became increasingly pronounced with increasing stages of fibrosis (2, 3, 4). Generally, significance levels of the AI-fibrosis scores were lower than the corresponding ‘classical image analysis’ approach (separation of the patient group with fibrosis score 0 from the groups with fibrosis scores > 0). This shows the higher resolution of the AI-based approach. This result reflects the way the different methods operate. In the ‘classical computer vision’-based determination of fibrosis, the relative area is quantified. However, this does not change a lot, until a severe stage of fibrosis is reached. In contrast the AI-based score is trained to recognize subtle fibrotic changes, just like a pathologist.

## Discussion

The deep learning-based method described here automates the widely used liver scores on ballooning, inflammation, steatosis, and fibrosis to quantify NAFLD/NASH progression in human liver biopsies. We are convinced that in the future, such AI methods will find their way into routine pathological grading of NAFLD/NASH (after independent diagnosis by a physician), provide improved readouts of clinical studies, support biomarker discovery and patient stratification, or enable the rapid assessment of the suitability of transplant organs.

Compared to NAFLD/NASH scoring from a human observer, our method has four main advantages.

First, it automates this routine task with a system that can run tirelessly. Thereby it reduces the workload of in-demand experts.

Second, it is designed to provide higher resolution scores than the discrete pathologist-based liver scores, while remaining interpretable for pathologists, since the range of scores is kept identical to the original score. This might help to quantify more subtle changes for a more refined disease assessment. For example, to show small reductions in fibrosis degrees, or reductions in the NAFLD activity score (NAS, sum of steatosis, inflammation, and ballooning scores). Importantly the AI-based NAS keeps the original interpretation and range familiar to pathologists^10^ but with the benefit of higher resolution. Depending on the therapeutic modality, a reduction in fibrosis score or in NAS could indicate disease improvement and serve as endpoint of a clinical trial. In combination with other patient data such as omics data, continuous tissue-based readouts might be also helpful for target identification, biomarker discovery and patient subgroup identification.

Third, unlike human observers who suffer from substantial inter- and intra-observer variability, the method will always provide reproducible results—anytime, everywhere.

Fourth, on the long run, deep learning-based systems continuously improve with added training data.

Ultimately, the last two aspects will lead to improved standardization of histological NASH/NAFLD readouts across investigators.

The major limitation of our method is that amount and variety of data are too small for good generalization to new data sets, and it will have a bias on the current training data. This can be seen in the lower performance metrics on our test data. For completely new data (e.g., from another laboratory), these metrics are expected to decrease further. Only relatively few individuals with completely healthy livers or very severe NAFLD/NASH (i.e., very high NAS scores) were represented in the dataset. This probably affects the performance of the approach at the lower and upper end of the scores (e.g., in case of ballooning, and inflammation where the current performance is only moderate).

These limitations can be remedied by increasing the number and variety of image tiles used for CNN training. Furthermore, adding more diverse patient data with a wider distribution of scores will be of benefit for the system’s performance. In general, the results of AI methods are dependent on the variability of the data with which they were trained. Therefore, to improve generalization ability (e.g., reduce the difference between training and test performance), it is necessary to add data from a variety of hospitals, laboratories, morphologies, and stains. Adding data incrementally can lead to an increasingly robust and accurate application that works reliably in a real-world environment.

The approach was built by following design objectives for easy reproducibility and extension. We made the source code and the training data open and public. Open-source deep learning libraries are used. That allows results replication, and extension of the method by anyone.

Future AI-based analysis approaches could for example capture isolated morphological alterations (e.g., bridging fibrosis, or isolated features of the substages of fibrosis score 1a-c), or analyze other features of interest (e.g., microsteatosis) and thereby move a step beyond mimicking scores designed for human observes. This can also help overcoming current caveats of the continuous scores: While for some features and grades continuous transitions are directly conceivable (e.g., ballooning, inflammation), for other features and grades this is not yet clear (e.g., certain transitions of fibrosis).

Our biopsy samples were of varying quality (i.e., stain quality, amount of liver tissue present). Such data is much more challenging for a model to fit than highly standardized clinical trial data^25^. At the same time, the heterogeneity in our dataset, with differences in the sample processing and staining as well as scanning procedure, more closely reflects real-world scenarios from routine hospital work. We thus emphasize that a model trained by diverse data has all the potential to lead to more robust applications.

These factors distinguish our method from two previously published methods^25,26^. Taylor-Weiner et al.^25^ use a proprietary tool and non-public data. The method^25^ is based on a segmentation approach and uses biopsy samples from clinical trials. For fibrosis, the score is provided in the range of the established fibrosis score^10,27^, whereas for the other three features of active injury (ballooning, inflammation, steatosis) area coverages are reported with a different range and unclear mapping to Kleiner’s NAS score components. Qu et al*.* use whole slide labels for training from a small dataset (N = 87)^26^, which is an interesting technology, but we assume would require very large datasets to perform well, like weakly supervised approaches^18^, where missing localized labels are compensated by very large training datasets.

For an unbiased quantitative comparison of approaches, an open test dataset of human liver biopsies along with pathologist’s scores will be required, which is not yet available.

In our opinion, future pathologists will be increasingly supported by automated tools. The pathologist's role will shift from manual analysis to informed use of AI systems and control of AI results. In this symbiotic combination, pathologists will achieve faster and more accurate results than before.

The method presented here is experimental and for research only. It is not approved for diagnostic use.

## Materials and methods

### Human liver biopsy microscopy images and ground truth scores

In total 467 clinically indicated human liver biopsies from 3 sources were used in this study in a retrospective analysis. Table 2 gives an overview on sources, digital slide scanners and stains.

**Table 2:** Overview on training data: sources, scanners, stains, and number of samples.

Source	Scanner	Stain	N biopsies
Duke	Aperio (Leica)	Masson Goldner	338
MHH	AxioScan Z1 (Zeiss)	Masson Trichrome, Masson Goldner	72
BI-int	AxioScan Z1 (Zeiss)	Masson Trichrome	57
Sum			467

338 of the scanned biopsies were obtained from the Duke University (Duke Department of Medicine, Durham, USA). For the Duke slides also the patient age was available. The experimental protocols were approved by an Institutional Review Board (Duke Health, DUHS IRB Office, Hock Plaza, Suite 405, 2424 Erwin Road, Durham, USA).

72 scanned biopsies were obtained from the Medizinische Hochschule Hannover (Institut für Pathologie, Hannover, Germany). The experimental protocols were approved by the ethics committee of the Medizinische Hochschule Hannover (Ethical vote no. 8667_BO_K_2019, Ethik-Kommission der MHH, OE 9515, Carl-Neuberg-Str. 1, 30625 Hannover, Germany).

57 scanned biopsies were obtained from internal repositories at Boehringer Ingelheims Biberach (Germany) and Ridgefield (USA) sites. Provider for the Boehringer Ingelheim samples was Discovery Live Sciences (Huntsville, AL, USA). The experimental protocols were approved by the Business Practice “Acquisition and Use of Human Biospecimens”, Discovery Research Coordination (Boehringer Ingelheim, Birkendorfer Str. 65, 88400 Biberach, Germany).

Written informed consent was obtained from the subjects and/or their legal guardian(s). The study conformed the ethical guidelines of the 1975 declaration of Helsinki.

Liver samples consisted of two types of hepatic biopsies: wedge biopsies and fine needle biopsies. Wedge biopsies contained a generous amount of material (typical dimension ~ 0.8 cm edge length) and numerous portal areas. Fine needle biopsies had a typical length of 1–2 cm and contained between 6 and 10 representative portal triads^28^.

Slides were either stained with the Masson Goldner or the Masson Trichrome stain according to established protocols. Whole slide microscopy images were acquired with a Leica Aperio scanner (Leica Biosystems, Wetzlar, Germany) or a Zeiss Axioscan Z1 scanner (Carl Zeiss, Jena, Germany) using a 20 × objective in bright field illumination.

The 467 biopsies were randomly split into two sets. Set 1 contained 296 biopsies. CNN training tiles from this set 1 were further split into train and validation sets (see below). Scoring artificial neural network fitting (see below) was performed on the whole set 1 using 4 × cross validation. Set 2 (test) contained 171 biopsies and was only used to evaluate the performance of the trained CNN.

Scoring per biopsy according to the features of Kleiner’s NAS score^10^ ballooning, inflammation, and the separated fibrosis grade^10^ was performed at the respective source site by a clinical pathologist specialized on NASH (338 samples from the Duke University and 72 samples from the Medizinische Hochschule Hannover) or a trained biomedical expert with 10 years of experience in NASH histopathology and Kleiner and Brunt scoring, for the 57 Boehringer Ingelheim samples. The scoring procedure per slide followed the established procedure described in detail by Kleiner^10^. Briefly the four hallmarks of NASH were defined and scored as follows:

1. Steatosis: This parameter refers to the amount of surface area involved by steatosis. Focus was set on vacuolar changes, where large and medium-sized lipid droplets (macrosteatosis) displace the nucleus and cell organelles to the cell periphery. The relative vacuole area coverage was determined automatically since scores of human experts were found to have a substantial systematic bias (see Supplementary Fig. S1). A CNN-based segmentation approach to recognize liver tissue and vacuoles was trained using Halo-AI (Indica Labs, Albuquerque, NM, USA), and steatosis scores were obtained using thresholds from^10^*:* 0 (< 5%), 1 (5–33%), 2 (> 33–66%), 3 (> 66% steatosis area). We however noticed that the threshold values of the Kleiner score combined with an accurate area determination resulted in a situation where the highest score (Steatosis score 3: > 66% Steatosis coverage) was not populated by any of our 467 biopsies—effectively resulting in a situation where the steatosis score only manifests between zero and two. This human-bias might require revising the steatosis scoring scheme and the area thresholds of Kleiner et al.^10^. At present, this could impair comparability and compatibility between pathologist- and AI-driven Kleiner scoring approaches.
2. Lobular inflammation: mononuclear cells as well as neutrophils were assessed and scored according to the overall assessment of all inflammatory foci into scores 0 (no foci), 1 (< 2 foci), 2 (2–4 foci), or 3 (> 4 foci per 200 × field) as defined in more detail in Ref.^10^.
3. Hepatocyte ballooning, hepatocytes with the morphological characteristics of hydropic degeneration (i.e., cytoplasmic vacuolation, cell swelling and rounding of a rarefied cytoplasm, and a central nucleus) were analyzed and assigned to scores 0 (none), 1 (few Ballooning cells), or 2 (many), as defined in Ref.^10^.
4. Fibrosis, the scoring was simplified according to the work Younossi^29^ with scores 0 (none), 1 (centrilobular/perisinusoidal fibrosis), 2 (Centrilobular and periportal fibrosis), 3 (Bridging fibrosis), 4 (Cirrhosis). The substages 1a, b, and c used in Ref.^10^ were not considered, as it was not suitable for our goal to obtain a single numeric score per feature.

Not for all biopsies all scores were obtained. A few slides with severe artifacts (e.g., very dark stain) were excluded from the analysis after manual inspection by B.S. In result, from the 467 slides, 453 had a steatosis score, 388 a ballooning score, 249 an inflammation score, and 384 a fibrosis score. Supplementary Table S1 shows an overview on the distribution of pathologist scores.

### Whole slide image preprocessing and tile generation

Whole slide images (WSI) were converted from the native format of the microscope vendor (e.g., czi, or ndpi) to TIFF or BigTIFF (for files over 4 GB) and scaled down to a resolution of 0.44 µm/px.

Next, the TIFFs were converted into adjacent, non-overlapping tiles using Halcon image processing software version 18.11 (https://www.mvtec.com/products/halcon, MVTec Software GmbH, Munich, Germany). Tiles of 299 px × 299 px were created at two dimensions: ‘high resolution tiles’ at 0.44 µm/px (ballooning, inflammation, steatosis) and ‘low resolution tiles’ after 1:3 downscaling to 1.32 µm/px (fibrosis).

### Tile annotation for CNN training

For ballooning, inflammation, steatosis, and fibrosis annotated datasets of tiles were created from slides of set 1. For each model, classes were defined, corresponding to relevant histopathological structures visible at a tile level, e.g., presence or absence of a ballooning cell. Figure 2 gives an overview on the models and classes. In case of ballooning, inflammation and fibrosis, an experienced biomedical expert with 10 years of experience in NASH pathology (B.S.) annotated the tiles. For steatosis, an automatic annotation based on the U-Net architecture^30^ and subsequent sorting based on fractional vacuole area coverage bins was chosen. Annotated tiles of the four models were randomly spit into 95% for CNN training and 5% for CNN validation. For full details on the morphological definition of the classes for ballooning, inflammation, and fibrosis, see supplementary material (section: Tile annotation and CNN models).

### CNN training and classification

CNNs for ballooning, inflammation, and steatosis were trained and validated on the annotated tiles. The Inception-V3 backbone in the Tensorflow—Keras implementation was used as CNN^31^, with a few modifications in the last layers. During application, the trained CNNs were applied to the corresponding tiles of the respective model (using ‘high resolution tiles’ for ballooning, inflammation, steatosis and using ‘low resolution tiles’ for fibrosis). For details, see supplementary material.

### Scoring-artificial neural network (ANN)

For each model (ballooning, inflammation, steatosis, and fibrosis), the results of all tiles belonging to one slide were aggregated by an ANN to obtain a single score per slide.

Numeric features describing the classification results of the distributed tiles on a given slide were generated and used as input of the ANNs. The ANNs were feedforward multilayer perceptrons with two hidden layers in regression mode to predict the ground truth (pathologist sores). The mean squared error (MSE) loss between pathologist score and continuous output of the ANNs was minimized. A custom activation function ensured that continuous ANN outputs remained in the range of the pathologist score, e.g., 0–4 for fibrosis. For details, see supplementary material.

### Image analysis of collagen content by color deconvolution and thresholding

The biopsy images from the Duke University were also analyzed for collagen content by ‘classical image analysis’, i.e., color deconvolution and subsequent thresholding to retrieve the color component of the Trichrome stain corresponding to collagen. For analysis, the digital pathology software HALO 3.2 was used (Indica Labs; Albuquerque, NM, USA). For details, see supplementary material.

## Supplementary Information

Supplementary Information.

## Data Availability

The datasets generated and/or analysed during the current study are available in the Open Science Framework repository, https://osf.io/8e7hd/. Source code of the method in the paper is available at GitHub, https://github.com/Boehringer-Ingelheim/deep-learning-based-quantification-of-NAFLD-NASH.
