# Supplementary material for: Deep learning-based quantification of NAFLD/NASH progression in human liver biopsies
Source: Sci Rep. 2022 Nov 10;12:19236. doi: 10.1038/s41598-022-23905-3 (PMC9649648; doi:10.1038/s41598-022-23905-3)
Supplement: Supplementary file 1 — Supplementary Information. [file 41598_2022_23905_MOESM1_ESM.docx]

**Deep learning-based quantification of NAFLD/NASH progression in human liver biopsies**

**Fabian Heinemann^1*^, Peter Gross^1^, Svetlana Zeveleva^2^, Hu Sheng Qian^2^, Jon Hill^3^, Anne Höfer^4^, Danny Jonigk**^4^**, Anna Mae Diehl**^5^**, Manal Abdelmalek^5^, Martin C. Lenter^1^, Steven S. Pullen^2^, Paolo Guarnieri^2^, Birgit Stierstorfer^1, 6^**

^1^ Drug Discovery Sciences, Boehringer Ingelheim Pharma GmbH & Co. KG, 88397 Biberach an der Riß, Germany

^2^ Cardiometabolic Diseases Research, Boehringer Ingelheim Pharmaceuticals, Inc., 900 Ridgebury Road, Ridgefield, CT, 06877 United States

^3^ Global Computational Biology and Digital Sciences, Boehringer Ingelheim Pharmaceuticals, Inc., 900 Ridgebury Road, Ridgefield, CT, 06877 United States

^4^ Institute of Pathology, Hannover Medical School, and the German Center for Lung Research (DZL), Biomedical Research in Endstage and Obstructive Lung Disease Hannover (BREATH), 30625 Hanover, Germany

^5^ Duke Department of Medicine, Gastroenterology, Lasalle Street, GSRB 1, Durham, NC 27710, United States

^6^ Non-Clinical Drug Safety, Boehringer Ingelheim Pharma GmbH & Co. KG, 88397 Biberach an der Riß, Germany

^*^ Corresponding author

**Supplementary Results**

***CNN transfer learning experiments and evaluation***

To find the most effective training approach, we compared three different transfer learning schemes for CNN training ^1^ (Fig. 3A). The first approach was training the fibrosis CNN model without any pre-training (i.e., without ImageNet-based CNN filters) and only training on the human liver data. Here an accuracy of 61.0% on the validation tiles was obtained. The second approach involved a model that was pre-trained on the ImageNet dataset and subsequently trained on the annotated human liver data. Here the accuracy improves to 79.4%, highlighting the strong impact of ImageNet pretraining ^2^. Generally, it is observed that pretraining on the huge ImageNet dataset helps creating general purpose convolutional filters ^3^. These powerful filters are also of benefit for other domains ^2^ such as histopathology, as shown by the increase in accuracy. Thus, moderately sized datasets like the one in this study (~ 10^3^-10^4^ image tiles) strongly benefit from pretraining on very large datasets like ImageNet. The third approach used existing CNN models to classify identical features of rodent NAFLD / NASH as model initialization and subsequently trained on the human data. We speculated that these CNNs trained on highly comparable rodent structures might help with the recognition of structures in human. Indeed, as seen in Fig. 3A the learning curves for the example case of fibrosis increased slightly faster (showing prior knowledge) and the overall validation accuracy was with marginally better with 80.3%. However, in all cases effects of using rodent pre-trained CNNs were rather minute and in practice might not justify the additional effort. For all four CNNs, the confusion matrix confirmed a very good classification performance of recognizing the relevant histological features (Fig. 3B shows the confusion matrix on the fibrosis validation data as an example). Most entries were found on the diagonal, displaying a high agreement between the results of the CNN and annotator. Deviations were almost exclusively with neighboring classes (e.g., +/- 1).

Summarized, we obtained the following classification accuracies for the CNN models on the validation data: Ballooning A = 98.0% (ImageNet and rodent ballooning pre-training, annotated tiles N = 54001, number of classes c = 3), Inflammation A = 79.1% (ImageNet and rodent inflammation pre-training, N = 10942, c = 4), Steatosis A = 69.4% (ImageNet pre- training, no suitable rodent model for initialization was available, N = 44581, c = 16), Fibrosis A = 80.3% (ImageNet and rodent fibrosis pre-training, N = 6704, c = 6).

**Investigation of a potential age-bias in pathologists scores**

We assumed that heterogeneity of the pathologist scores in the ground truth provided a particular challenge for the ANN fitting. This heterogeneity was previously shown by high intra- and inter-annotator variabilities of pathologists in the scoring task.

To reduce heterogeneity in the ground truth, we hypothesized that pathologists might assign higher scores to patients who they knew are older (e.g., the same type of histopathological alteration was scored higher under the prior knowledge of higher age). For this purpose, we compared the difference of the pathologists scores with the unbiased AI scores on the test dataset. In case of an age bias, this difference should change with age. However, as shown in Fig. S3 our analysis revealed that surprisingly no notable age-bias of the pathologist’s scores could be found.

**Supplementary Materials and Methods**

***Tile annotation and CNN models***

The four CNN models for ballooning, inflammation, steatosis, and fibrosis were trained on an annotated dataset consisting of image tiles from the training set. For each model, suitable classes were defined, corresponding to relevant histopathological structures visible at a tile level, e.g., presence or absence of a ballooning cell.

Figure 2 gives an overview on the models and classes per model. In case of ballooning, inflammation and fibrosis, an experienced biomedical expert with 10 years of experience in NASH pathology (B.S.) annotated the tiles by assigning them corresponding to predefined classes for each model. For steatosis, an automatic annotation was chosen. We employed an ad-hoc semantic segmentation tool in python, based on the U-Net architecture, and utilized it to quantify the factional area coverage.

Depending on fraction of area covered by steatotic vacuoles (macro-vesicular steatosis) tiles were placed into bins (specifically, moved in folders) for a total of fifteen classes. The first bin was populated with tiles between 0% to 5% area coverage. The area coverage for each bin increased in 5% steps, with a constant bin width of 5%. We used 4000 tiles per bin, up until a bin interval of 40% to 45%. Beyond this point the number of samples per bin decreased, with the minimum falling between 70% to 75% (N=4). This decline in samples per bin originates from the fact that very high fractional coverage of steatotic vacuoles is rare. Subsequent human curation ensured removal of false positives. The ‘ignore’ class was again defined as described above. In total 44581 annotated tiles were available for the steatosis model (for details about the distribution of classes see Table S2).

In the case of ballooning, these classes were ‘0’ (i.e., no ballooning cell present), ‘1’ (i.e., ballooning cell present), and ‘ignore’ (less than 50% tissue on tile, artifacts, such as out of focus, no liver tissue). In total 54001 tiles from set 1 were annotated for the ballooning model (for details about the distribution of classes see Table S3).

For inflammation, we used the classes ‘0’ (background class with no marked inflammation, defined as absence of clusters with more than two inflammatory cells and less than five disseminated inflammatory cells per tile), ‘1’ (cell clusters between three and five inflammatory cells and/or between five and ten disseminated inflammatory cells per tile), ‘2’ (a cluster of more than five inflammatory cells and/or more than ten disseminated inflammatory cells visible per tile), and ‘ignore’ (defined as above). In total 10942 tiles from set 1 were annotated for the inflammation model (for details about the distribution of classes see Table S4).

For fibrosis, the classes for the per-tile classification correspond to the macroscopic definition of the fibrosis score defined by Brunt. The ‘ignore’ class was defined as described above. In total 6704 tiles from set 1 were annotated for the fibrosis model (for details about the distribution of classes see Table S5).

Annotated tiles were randomly spit into 95% for CNN training and 5% for CNN validation.

***Tile label tool***

A part of the training was performed using a custom developed software, LabelTool (Fig. S2). It allowed to visualize classifications of CNN models, and to annotate tiles within the context of the surrounding tissue by clicking within a depiction of a slide. The visualization of current model’s classifications allowed for example to train specifically on weaknesses of a current model (e.g., classification errors for specific histological structures). This approach is inspired by the ‘active learning’ paradigm and helped to perform time-efficient training with visual feedback for the annotating person, instead of blindly annotating tiles. Software development was done by the Machine Vision Makers (<http://www.machine-vision-makers.com/>, Munich, Germany).

***CNN training and classification***

We experimented with three distinct pretraining schemes: a) no pretraining, b) pretraining on ImageNet, c) pretraining on ImageNet and similar data. In the latter case, the training was initialized on a CNN that was trained to classify the equivalent rodent features while utilizing ImageNet weight initialization.

We made the following modifications compared to the original Inception-V3 implementation: The last fully connected layer was removed, and replaced by a global average pooling layer, followed by dropout to avoid overfitting ($p=0.5$). The last layer was a fully connected layer with Softmax normalization, where the number of outputs corresponded to the number of classes for each model.

CNN training was performed with stochastic gradient descent with momentum ($\mu=0.9$). During training, the categorical cross entropy loss between the model prediction and the ground truth was reduced on the validation set, which was not used to update weights of the CNN. 5% of annotated tiles from set 1 were split off into the validation set randomly for that purpose. A learning rate scheduler was used with an initial learning rate of $\eta$ = 0.5 10^-4^. The learning rate was reduced by a factor of 5 if the validation loss did not decrease for more than two epochs. The minimal learning rate was $\eta=$ 10^-7^.

Class imbalances were compensated by weighting the sampling process with $n/n_{i}$, where $n$ is the total number of training examples and $n_{i}$ the number of examples for class $i$.

The robustness of all four CNNs against rotation and translation was improved via augmentations. Random rotations in the range $\theta\epsilon\left[ -\frac{\pi}{4},\frac{\pi}{4} \right]$, translation in x and y by up to 15%, and horizontal and vertical flipping were applied.

The four CNN models were applied to the whole slide images after they were converted into tiles. CNNs for ballooning, inflammation, and steatosis were applied to the ‘high resolution tiles’ (0.44 µm/px) whilst the CNN for fibrosis was applied on the ‘low resolution tiles’ (1.32 µm/px).

The Softmax output of all four CNNs was further processed as follows. The Softmax output is a vector $\left( q_{0} \ldots q_{C-1} q_{C} \right)^{T}$ with confidence values for the classes of each model with ‘ignore’ as class *C*. Only tiles where ‘ignore’ was not the predicted class where further processed (i.e., $q_{C} \neq arg max\left( q_{0} \ldots q_{C-1} q_{C} \right)^{T}$). To correct confidences for cases where a tile contained a fraction of ‘ignore’ (e.g., a tissue edge visible on a tile but also a ballooning cell), which will reduce the respective output confidences for the relevant class (e.g., ballooning), the confidences $q$ where re-normalized to a sum of 1 without ‘ignore’, yielding the normalized confidences $p$:

$$p_{j}= \frac{q_{j}}{\left( \sum_{i=0}^{C-1} q_{i} \right)}$$

***Scoring-Artificial Neural Network (ANN)***

For each model (ballooning, inflammation, steatosis, and fibrosis), the results of all tiles belonging to one biopsy were aggregated by an ANN to obtain a single score per slide. The ANNs were trained against the ground truth from the pathologist. Since these four ANNs output a single number per biopsy (i.e., the continuous Kleiner & Brunt score), we also refer to them as scoring ANNs.

We used the following features for a model of $c$ classes (without ‘ignore’) and N tiles, all originating from the output of the CNNs, as input of the scoring ANNs (see Fig. 5):

1. The average weighted class per slide: $x=\frac{1}{N}\sum_{liver} \sum_{i=0}^{c-1} ip_{i}$. Here $i$ are the numerical values of the respective classes and $p_{i}$ the normalized confidences (e.g.: for fibrosis $c=5$: $i$ = 0, 1, 2, 3, 4 for the fibrosis classes ‘0’, ‘1’, ‘2’, ‘3’, ‘4’).
2. The Shannon entropy for each class $i$ per slide (excluding ‘ignore’): $S_{i}=-\sum_{liver} p_{i}log{(p}_{i})$
3. And the average score for each class $i$ per slide (excluding the class ‘ignore’, see above): $\left\langle p_{i} \right\rangle=\frac{1}{N}\sum_{liver} p_{i}$

These aggregated features were standardized by subtracting the mean and dividing by the standard deviation of set 1 (see: Human liver biopsy samples and ground truth scores). These standardized features served as input for the scoring-ANNs of the respective model. The scoring ANNs are feedforward multilayer perceptrons, comprising the following structure: an input layer with $d$ inputs connected to the first hidden layer with 100 nodes ($d$ = number of computed features per slide). We used exponential linear unit (eLU) ^4^ as activation function of the first hidden layer, which empirically resulted in better fits. A dropout of $p=0.8$ was applied during training to avoid overfitting. The second hidden layer also comprised 100 nodes, combined with a Rectified linear unit (ReLu) activation function. The output neuron used a custom activation function to ensure that the scores remained in the predefined range of the pathologist scores for the respective model, i.e., 0-2 for ballooning, 0-3 for inflammation, 0-3 for steatosis, and 0-4 for fibrosis. For an output $u$of the last neuron, a maximum and a minimum integer pathologist score of $s_{max}, s_{min}$the resulting score $s$ was computed as follows:

$$s=s_{min}+ \frac{1}{2}\left( \tanh\left( u \right)+1 \right)\left( s_{max}-s_{min} \right)$$

This custom activation function keeps the (continuous) result in the range $s \epsilon\left[ s_{min}, s_{max} \right]$, i.e., the range of the pathologist score.

The ANNs were trained for 1000 epochs in a 4-fold cross validation loop. An NAdam ^5^ optimizer with an initial learning rate of 0.0003 was used. During training, the learning rage was reduced by 10% if the validation loss did not decrease for more than two epochs up to a minimal learning rate of 10^-8^. The mean squared error (MSE) loss between the ANN score and pathologist score was used as loss during training.

***Class activation maps***

Class activation maps were generated according to the method described by Zhou et al. ^6^. Briefly, the approach works as follows: The last convolutional layer contains spatial information which can be utilized to highlight the area most relevant for a classification. The last convolutional layer is a tensor of dimension $\left( f_{kxy} \right)$ 2048 x 8 x 8, with $\left| k \right|=2048$ feature maps (filtered image properties) and a spatial resolution $x, y$ of 8 x 8. The following global average pooling layer averages the features spatially: $F_{k}=\sum_{x,y} f_{kxy}$ (note: the constant denominator $\frac{1}{\left| k \right|}$ is omitted for simplicity, see ^6^). Next, the output $s_{c}$for class $c$ is computed using the last fully convolutional layer with weights $w_{ck}$ (note: the bias term is omitted for simplicity as well, see ^6^). This can be written as: $s_{c}=\sum_{k} w_{ck}\sum_{x,y} f_{kxy}$, or $s_{c}=\sum_{x,y} \sum_{k} w_{ck}f_{kxy}= \sum_{x,y} M_{c}(x,y)$. The term $M_{c}\left( x,y \right)= \sum_{k} w_{ck}f_{kxy}$ can by computed for each $x, y$ location and thus corresponds to a spatially resolved activation map for class $c$. By scaling the 8 x 8 activation map $M_{c}\left( x,y \right)$ to the size of the input tile, e.g., 299 x 299, activated areas for a decision for a class $c$ are highlighted.

***Image analysis of collagen content by color deconvolution and thresholding***

The biopsy images from the Duke University were also analyzed for collagen content by ‘classical image analysis’, i.e., color deconvolution and subsequent thresholding to retrieve the color component of the Trichrome stain corresponding to collagen. 193 whole slide digital images from set 1 were imported into HALO image analysis software (Indica Labs; Albuquerque, NM, USA) and annotated. Slides containing multiple serial sections were distinguished as separate annotation layers and mean values were reported. A custom algorithm was prepared using the Halo Area Quantification v2.1.7 module. Colors representing collagen, and non-fibrotic liver tissue were assigned for deconvolution. Pixel thresholding values were assigned to determine percent area of Masson Goldner stain for collagen deposition. Most artifacts, mucin, and other extracellular matrix components were excluded. Quantitative data was grouped relative to Brunt’s fibrosis score (0-4). Statistical analysis was performed using the software GraphPad Prism 9 (GraphPad Software, San Diego, Ca, USA).

**
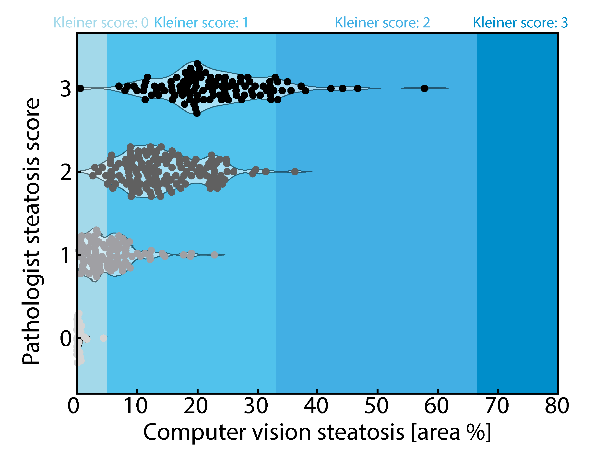
**

**Fig. S1:** **Comparison of the pathologist’s steatosis score with the quantification of the area fraction covered by steatotic vesicles, based on computer vision.** Each dot represents a slide, which was analyzed by a pathologist and by computer vision in parallel. The blue background colors highlight the regions of steatotic area coverage that correspond to the Kleiner steatosis score. A substantial over-estimation of the pathologist-score is apparent, highlighting the difficulty for humans to estimate area from an image.


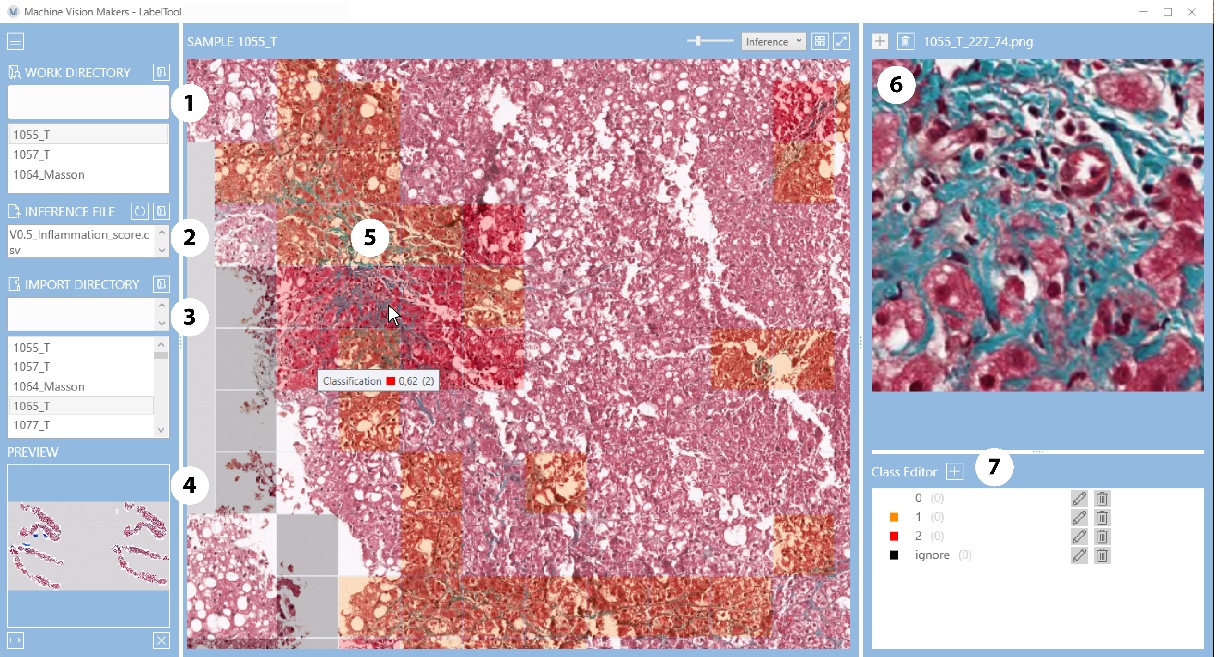


**Fig. S2:** **Custom LabelTool for training and result inspection in the context of the whole slide image**. This tool allows to annotate tiles in the context of the whole slide. The work directory (1) allows selecting biopsy images. To show results of a previous classification (2) an inference file can be selected. To prepare tiles for processing with the label tool, a directory containing tiles can be selected (3). The preview (4) shows tiles stitched to an image ready for import. The central main area (5) shows the result of the current classification (inference) indicated by colors. The area at the top right (6) shows the currently selected tile. The tool allows switching between three visualization modes: showing annotation labels, showing classification results, or showing a combination of labels and classification results. The last option can show deviations of current CNN classifications and pathologist’s annotations, which allows to annotate in a feedback loop (‘active learning’). The area at the bottom right (7) allows do select or define classes, which can be assigned by clicking to the area in the main window.


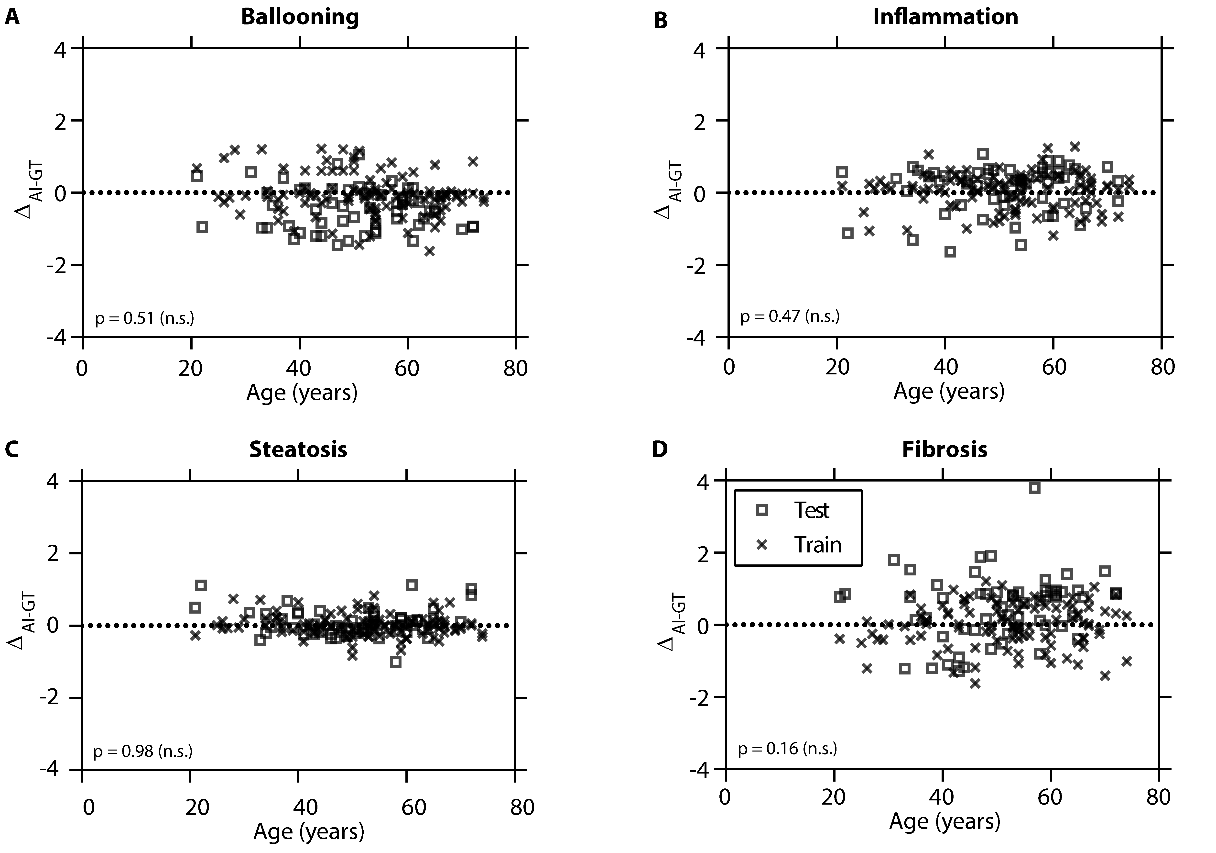


**Fig. S3:** **Investigation of a potential age bias in ground truth scores.** Plot of the difference Δ_AI_-_GT_ = s_AI_ – s_GT_ of the AI scores s_AI_ and the ground truth s_GT_ compared to the age of the patients. For **A)** ballooning, **B)** inflammation, **C)** steatosis, and **D)** fibrosis the ground truth was either the pathologist score or in case of steatosis a separate segmentation algorithm (see Materials & Methods). The AI score has no prior information about the patient age. Further, the AI score on the unseen test set was not used for training. Therefore, the AI score on test is an unbiased quantification of the state of the biopsy with respect to the four features investigated. In case of a potential bias of the ground truth, e.g., higher scores for older patients, the term Δ_AI_-_GT_ should change, since the potentially biased ground truth would deviate increasingly from the unbiased AI score. As seen in the graphs, all deviations Δ_AI_-_GT_ scatter around 0 without dependency on the age. Slopes of linear fits to the data on test were close to zero. P values in the graphs correspond to significance tests for the null hypothesis that the slope is non-zero. In all cases this hypothesis was rejected. Thus, no evidence for an age bias of the ground truth could be observed.

| Source | Steatosis | | | | Ballooning | | | Inflammation | | | | Fibrosis | | | | |
| --- | --- | --- | --- | --- | --- | --- | --- | --- | --- | --- | --- | --- | --- | --- | --- | --- |
|  | **0** | **1** | **2** | **3** | **0** | **1** | **2** | **0** | **1** | **2** | **3** | **0** | **1** | **2** | **3** | 4 |
| Duke | 99 | 221 | 6 | - | 30 | 112 | 128 | 2 | 126 | 42 | 4 | 28 | 60 | 95 | 73 | 14 |
|  |  |  |  |  |  |  |  |  |  |  |  |  |  |  |  |  |
| MHH | 15 | 49 | 6 | - | 3 | 39 | 19 | 2 | 39 | 5 | 1 | 15 | 15 | 14 | 14 | 13 |
|  |  |  |  |  |  |  |  |  |  |  |  |  |  |  |  |  |
| BI-int. | 53 | 4 | 0 | - | 52 | 0 | 5 | 11 | 6 | 5 | 6 | 12 | 0 | 18 | 3 | 10 |
|  |  |  |  |  |  |  |  |  |  |  |  |  |  |  |  |  |
| Sum | **167** | **274** | **12** | **-** | **85** | **151** | **152** | **15** | **171** | **52** | **11** | **55** | **75** | **127** | **90** | 37 |

**Table S1:** Overview of the distribution of liver score values for the biopsies. Scores correspond to the Kleiner & Brunt scoring system with the separated fibrosis score (without subtypes a, b, c). Not for all biopsies all scores were obtained or remained after quality control to sort out low quality samples.

| Class | N (Train) | N (Val) |
| --- | --- | --- |
| ‘0’ (0-5%) | 3812 | 188 |
| ‘1’ (5-10%) | 3792 | 208 |
| ‘2’ (10-15%) | 3810 | 189 |
| ‘3’ (15-20%) | 3790 | 209 |
| ‘4’ (20-25%) | 3797 | 202 |
| ‘5’ (25-30%) | 3787 | 207 |
| ‘6’ (30-35%) | 3776 | 215 |
| ‘7’ (35-40%) | 3778 | 199 |
| ‘8’ (40-45%) | 3749 | 209 |
| ‘9’ (45-50%) | 2878 | 154 |
| ‘10’ (50-55%) | 1125 | 54 |
| ‘11’ (55-60%) | 328 | 24 |
| ‘12’ (60-65%) | 75 | 3 |
| ‘13’ (65-70%) | 16 | 3 |
| ‘14’ (70-75%) | 3 | 1 |
| ‘ignore’ | 3809 | 191 |

**Table S2: Overview about annotated tiles for the steatosis model.** Total number of annotated tiles N = 44581.

| Class | N (Train) | N (Val) |
| --- | --- | --- |
| ‘0’ | 28552 | 1505 |
| ‘1’ | 796 | 54 |
| ‘ignore’ | 21900 | 1194 |

**Table S3: Overview about annotated tiles for the ballooning model.** Total number of annotated tiles N = 54001.

| Class | N (Train) | N (Val) |
| --- | --- | --- |
| ‘0’ | 6933 | 341 |
| ‘1’ | 1414 | 80 |
| ‘2’ | 1385 | 70 |
| ‘ignore’ | 675 | 44 |

**Table S4: Overview about annotated tiles for the inflammation model.** Total number of annotated tiles N = 10942.

| Class | N (Train) | N (Val) |
| --- | --- | --- |
| ‘0’ | 3486 | 193 |
| ‘1’ | 563 | 29 |
| ‘2’ | 326 | 23 |
| ‘3’ | 338 | 19 |
| ‘4’ | 386 | 24 |
| ‘ignore’ | 1275 | 60 |

**Table S5: Overview about annotated tiles for the fibrosis model.** Total number of annotated tiles N = 6704.

***Supplementary references***

1. Shaha, M. & Pawar, M. Transfer Learning for Image Classification. *2018 Second Int Conf Electron Commun Aerosp Technology Iceca* 00, 656–660 (2018).

2. Kornblith, S., Shlens, J. & Le, Q. V. Do Better ImageNet Models Transfer Better? *2019 Ieee Cvf Conf Comput Vis Pattern Recognit Cvpr* 00, 2656–2666 (2019).

3. Qin, Z., Yu, F., Liu, C. & Chen, X. How convolutional neural networks see the world --- A survey of convolutional neural network visualization methods. *Math Found Comput* 1, 149–180 (2018).

4. Clevert, D.-A., Unterthiner, T. & Hochreiter, S. Fast and Accurate Deep Network Learning by Exponential Linear Units (ELUs). *Arxiv* (2015).

5. Dozat, T. Incorporating Nesterov momentum into adam. *ICLR 2016* (2015).

6. Zhou, B., Khosla, A., Lapedriza, A., Oliva, A. & Torralba, A. Learning Deep Features for Discriminative Localization. *2016 Ieee Conf Comput Vis Pattern Recognit Cvpr* 2921–2929 (2016) doi:10.1109/cvpr.2016.319.
